# Formalin fixation increases deamination mutation signature but should not lead to false positive mutations in clinical practice

**DOI:** 10.1371/journal.pone.0196434

**Published:** 2018-04-26

**Authors:** Leah M. Prentice, Ruth R. Miller, Jeff Knaggs, Alborz Mazloomian, Rosalia Aguirre Hernandez, Patrick Franchini, Kourosh Parsa, Basile Tessier-Cloutier, Anna Lapuk, David Huntsman, David F. Schaeffer, Brandon S. Sheffield

**Affiliations:** 1 Contextual Genomics Incorporated, Vancouver, British Columbia, Canada; 2 Anatomical Pathology, Department of Pathology and Laboratory Medicine, University of British Columbia, Vancouver, British Columbia, Canada; CNR, ITALY

## Abstract

Genomic analysis of cancer tissues is an essential aspect of personalized oncology treatment. Though it has been suggested that formalin fixation of patient tissues may be suboptimal for molecular studies, this tissue processing approach remains the industry standard. Therefore clinical molecular laboratories must be able to work with formalin fixed, paraffin embedded (FFPE) material. This study examines the effects of pre-analytic variables introduced by routine pathology processing on specimens used for clinical reports produced by next-generation sequencing technology. Tissue resected from three colorectal cancer patients was subjected to 2, 15, 24, and 48 hour fixation times in neutral buffered formalin. DNA was extracted from all tissues twice, once with uracil-N-glycosylase (UNG) treatment to counter deamination effects, and once without. Of note, deamination events at methylated cytosine, as found at CpG sites, remains unaffected by UNG. After extraction a two-step PCR targeted sequencing method was performed using the Illumina MiSeq and the data was analyzed via a custom-built bioinformatics pipeline, including filtration of reads with mapping quality <30. A larger baseline group of samples (n = 20) was examined to establish if there was a sample performance difference between the two DNA extraction methods, with/without UNG treatment. There was no statistical difference between sequencing performance of the two extraction methods when comparing read counts (raw, mapped, and filtered) and read quality (% mapped, % filtered). Analyzing mutation type, there was no significant difference between mutation calls until the 48 hour fixation treatment. At 48 hours there is a significant increase in C/G->T/A mutations that is not represented in DNA treated with UNG. This suggests these errors may be due to deamination events triggered by a longer fixation time. However the allelic frequency of these events remained below the limit of detection for reportable mutations in this assay (<2%). We do however recommend that suspected intratumoral heterogeneity events be verified by re-sequencing the same FFPE block.

## Introduction

Current histology practice employs preservation of tissue by fixation in neutral buffered formalin prior to embedding in paraffin (Formalin Fixation Paraffin Embedded tissue; FFPE). Tissue preservation occurs due to the chemical reaction of formalin with nucleotides and proteins present in the tissue. A consequence to this chemical reaction is the occasional deamination of cytosine residues. Should this chemically modified DNA be replicated for amplification in the laboratory, the deamination site would be transitioned to a thymine nucleotide (C->T) resulting in a perceived mutation [1–4].

For next-generation sequence (NGS) based assays, it has been speculated that a deamination event in the tissue could be propagated through PCR amplification and result in the clinical reporting of a false positive due to the sensitive nature of NGS technology. Though several studies have reported that these artefacts are detected in research samples at low allelic frequency [5–8] few studies have explored the clinical significance of these events.

Although formalin fixation and paraffin embedding may be suboptimal for molecular studies, it is firmly entrenched in pathology practice. Rather than seeking alternative fixatives, successful clinical molecular laboratories must be able to work with FFPE material. This study examines the effects of pre-analytic variables introduced by routine pathology processing on specimens used for clinical next-generation sequencing studies.

## Materials and methods

### Study design

Three colorectal patient groups were used to establish if different sample treatments would adversely affect patient sequencing results: 1. A fixation group to determine formalin effects on tissues over fixation time, 2. A larger baseline group was used to establish baseline formalin effects when fixation time is uncontrolled, and 3. A block age group to determine age effects on patient sequencing results.

**Fig 1:**
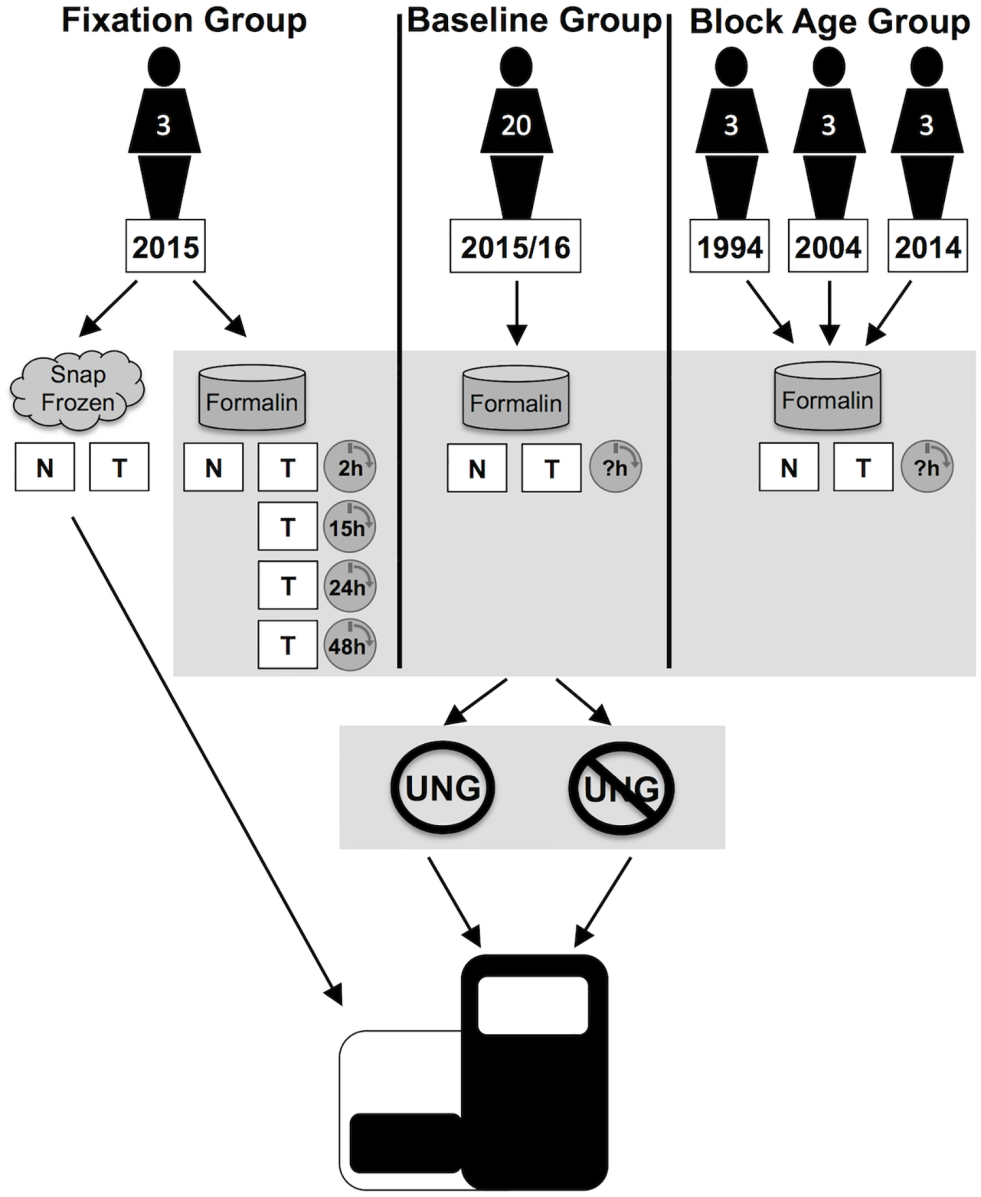
Experimental workflow. DNA extracted from three patient groups was sequenced after treatment with and without UNG. The fixation group consisted of three patients from 2015 where paired tissue samples were snap frozen or put into formalin for a determined length of times. A baseline group consisting of 20 patients all from 2015/16 were fixed in formalin for an unknown amount of time. The block age group consisted of three patients from 1994, three from 2004 and three from 2014; all samples had an unknown fixation time. DNA was extracted from normal (N) and tumor (T) tissue when available.

To establish if deamination effects could be mitigated enzymatically, the DNA extracted from each formalin fixed sample in all groups was subjected to uracil-N-glycosylase (UNG) treatment in parallel with tissues without UNG treatment as a control. The uracil-DNA glycosylase will cleave out the uracil from a DNA strand leaving an abasic site that will not be replicated. To ascertain mutation status, the DNA from all sample types was sequenced using the Illumina MiSeq. See workflow Fig 1.

### Patient samples for fixation group

This study was approved by the research ethics board of the Vancouver General Hospital (H15-00425) and was performed as part of ongoing quality assurance for clinical molecular testing. Thus direct consent from patients was not required as per institutional policy. A sequential set of cases of colonic adenocarcinoma were prospectively identified from the anatomical pathology lab of the Vancouver General Hospital in 2015. Inclusion criteria consisted of: colon adenocarcinoma undergoing surgical resection, and sufficient tumor mass for routine surgical pathology as well as five additional sections for study. Additionally, cases were only included if sufficient material was available to contribute to the study without conceivably compromising clinical care in any fashion. Cases were excluded based upon insufficient tumor mass and not directly meeting the other inclusion criteria. Metadata for the patients selected is listed in Table 1.

**Table 1:** Fixation group patient metadata.

Patient	Site	Stage	Max Tumor Dimension	Grade	Routine Genetics
1	Ascending	pT4a pN2a	8.0cm	Low	MSH2 deleted
2	Transverse	pT3 pN0	7.5 cm	Low	BRAF V600E (IHC), MMR intact
3	Sigmoid	pT3 pN0	7.0 cm	High	MMR Intact

Five similarly sized pieces of tumor were taken from each case for fixation. To establish ground truth mutation status, a single section was snap frozen in liquid nitrogen and then stored at -80°C. To establish formalin fixation effects, the remaining cases were fixed in neutral buffered formalin for 2, 15, 24, and 48 hours. Following fixation, blocks underwent standard clinical processing into paraffin blocks. The matched normal tissue underwent formalin fixation and all other procedures identical to those used in routine clinical practice at the pathology laboratory of a large academic center. Pathologist assessment of the tumor samples by hematoxylin and eosin (H&E) showed all the samples to be equal and representative in terms of grade, histologic subtype, cellularity, and tumor content.

### Additional patient groups

#### Baseline group

A series of 22 colorectal adenocarcinomas from resection specimens between 2015–2016 were identified and retrieved from the Vancouver General Hospital archives. All cases resected were reviewed by a pathologist (BTC) to confirm the diagnosis and assess tumor content. Twenty cases were finally selected based on sufficient tumor content (>10%). These were extracted with and without UNG treatment to establish a baseline for expected deamination signature.

#### Block age group

Three FFPE patient samples from 1994 three FFPE patient samples from 2004, and three patient samples from 2014 were extracted with and without UNG treatment to determine if block age had an effect on deamination events.

### DNA processing and sequencing

Genomic DNA was extracted from frozen samples using the Qiagen Gentra Puregene Kit as per manufacturer’s recommendations. FFPE DNA was extracted from FFPE tissues using the Qiagen QiaAmp FFPE Tissue Kit as per manufacturer’s recommendations except deparaffinization was performed using 90°C heat in mineral oil. To ascertain deamination effects on FFPE DNA, the Qiagen Gene Read Kit, which includes a UNG treatment step to eliminate DNA strands containing uracil, was employed as per manufacturer’s instructions. For direct comparison of UNG effects to non-UNG treatment, the Qiagen Gene Read kit was employed in both DNA extraction methods however UNG was withheld from the column for the non-UNG treatment.

DNA was prepared for sequencing using a two-step PCR method for the targeted regions listed in Table 2. The Nextera XT index kit was used to add Illumina specific adapters and barcodes for sample multiplexing onto one run. Pooled samples were loaded onto the Illumina MiSeq instrument and 151 bp paired-end reads were sequenced in parallel. All samples were run in triplicate.

**Table 2:** Targeted regions of amplification for the NGS assay.

Gene	Hotspot	Transcript	Gene	Hotspot	Transcript
*AKT1*	E17	NM_001014432.1	*IDH1*	R132	NM_005896.3
*ALK*	T1151, L1152, C1156, F1174, L1196, G1269, R1275	NM_004304.4	*IDH2*	R140, R172	NM_002168.3
*AR*	S741, W742, H875, Q876, T878	NM_000044.3	*JAK1*	V658, S703	NM_002227.2
*BRAF*	Q201, G466, G469, Y472, D594, G596, L597, V600	NM_004333.4	*KIT*	D816, D820, N822, Y823, exons 11, 13	NM_000222.2
*CDKN2A*	R58	NM_000077.4	*KRAS*	G12, G13, Q61, K117, A146	NM_004985.4
*CTNNB1*	S37, T41, S45	NM_001904.3	*MAP2K1*	Q56, K57, K59, D67, P387	NM_002755.3
*EGFR*	exons 18, 19, 20, 21	NM_005228.3	*MAP2K2*	F57, Q60, K61, L119	NM_030662.3
*ERBB2*	G309, S310	NM_004448.3	*MET*	Y1253, exons 13, 18	NM_001127500.1
*ESR1*	V534, P535, L536, Y537, D538	NM_001122742.1	*NRAS*	G12, G13, Q61, K117, A146	NM_002524.4
*FGFR1*	N546, K656	NM_023110.2	*PDGFRA*	D842	NM_006206.4
*FGFR2*	S252, P253, N549, K659	NM_000141.4	*PIK3CA*	E542, E545, Q546, D549, M1043, N1044, A1046, H1047, G1049	NM_006218.2
*GNA11*	Q209	NM_00267.4	*PTEN*	R130, R173, R233	NM_000314.4
*GNAQ*	Q209	NM_002072.4	*RET*	C634, M918	NM_020975.4
*GNAS*	R201	NM_000516.4	*STK11*	Q37, P281, F354	NM_000455.4
*HRAS*	G12, G13, Q61	NM_005343.2			

### Bioinformatic analysis

Sequence reads were aligned to the reference sequence (Genome Build HG19) using BWA [9] and results filtered to remove all aligned reads with four or more mismatching bases or six or more soft clipped bases. Single nucleotide variants (SNV) were identified and annotated using MutationSeq v4.3.8 [10] in paired deep mode with criteria -v -q 30—coverage 100 -t 0.5. The complete list of SNVs for all patient groups, is included in S1 Table. Reported SNVs were then extracted from the resulting VCF file with acceptance criteria read quality score of ≥30, probability score of ≥0.90, and coverage ≥500.

Basic statistics of total reads, and reads mapped were generated using bespoke scripts in Python including the packages BioPython and Pysam and plots drawn using Matplotlib. To compare read counts between three discrete groups the Kruskal-Wallis test was performed, and comparison over time was performed using linear regression, both using the Python scipy package.

To calculate deamination events counts of SNV calls from the MutationSeq VCF output was binned by reference and alternative bases, and deamination transitions (C→T, G→A) compared against all other substitutions (transversions). Since deamination events often occur at low allele frequencies, SNVs passing acceptance criteria at any variant allele frequency (VAF) % were included.

To limit analyses to only mutations introduced by the treatment method the flash frozen tumor samples from each patient were used as the paired normal sample for MutationSeq where available (the fixation group). In groups where flash frozen controls were not available paired normal samples were used to exclude somatic mutations.

## Results

### Characterization of sequencing QC metrics and deamination events in a baseline group of samples

**Fig 2:**
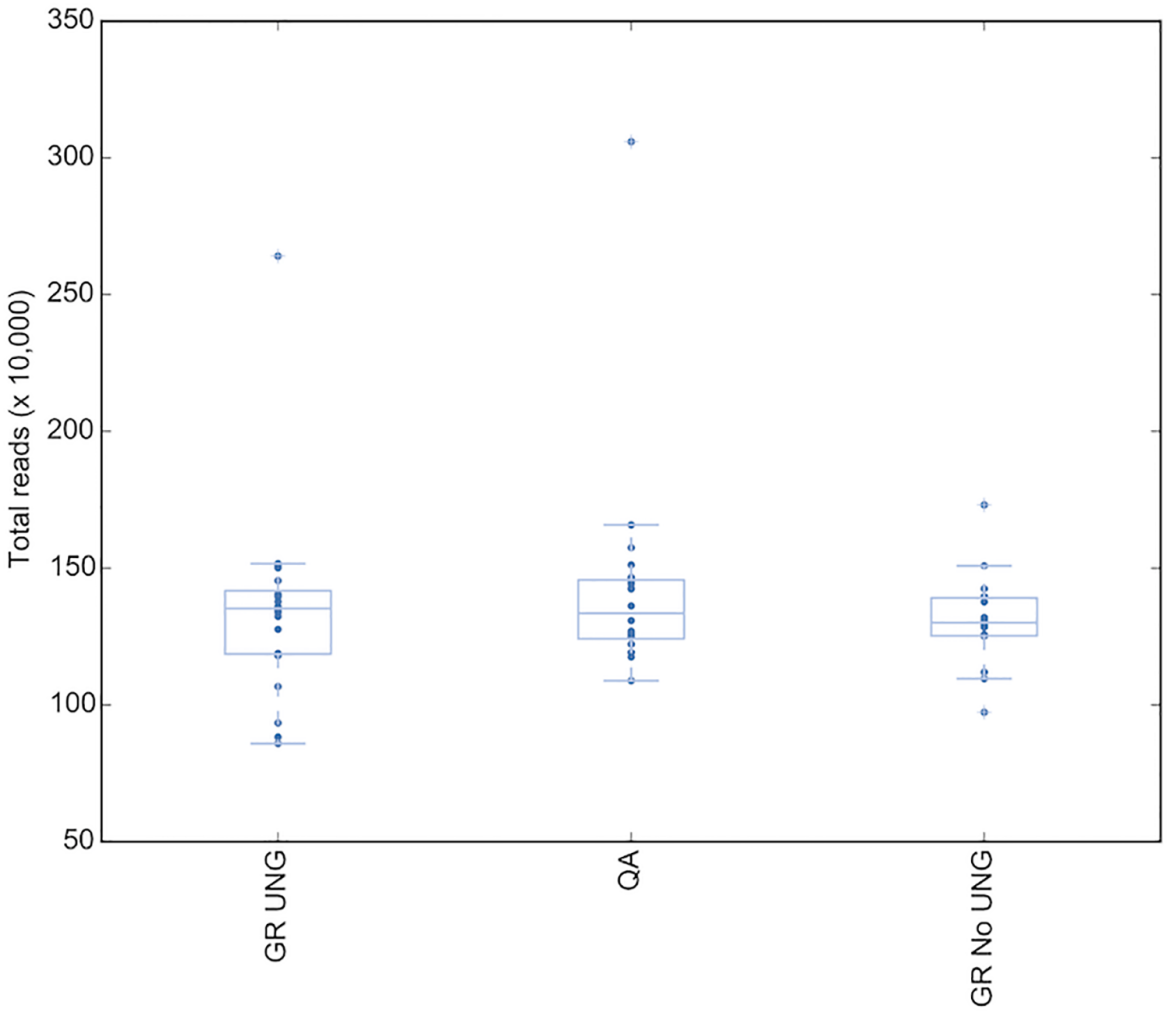
Total reads mapped in the baseline group does not change by DNA extraction type. GR UNG = GeneRead uracil-N-glycosylase, QA = QiaAmp FFPE kit.

**Fig 3:**
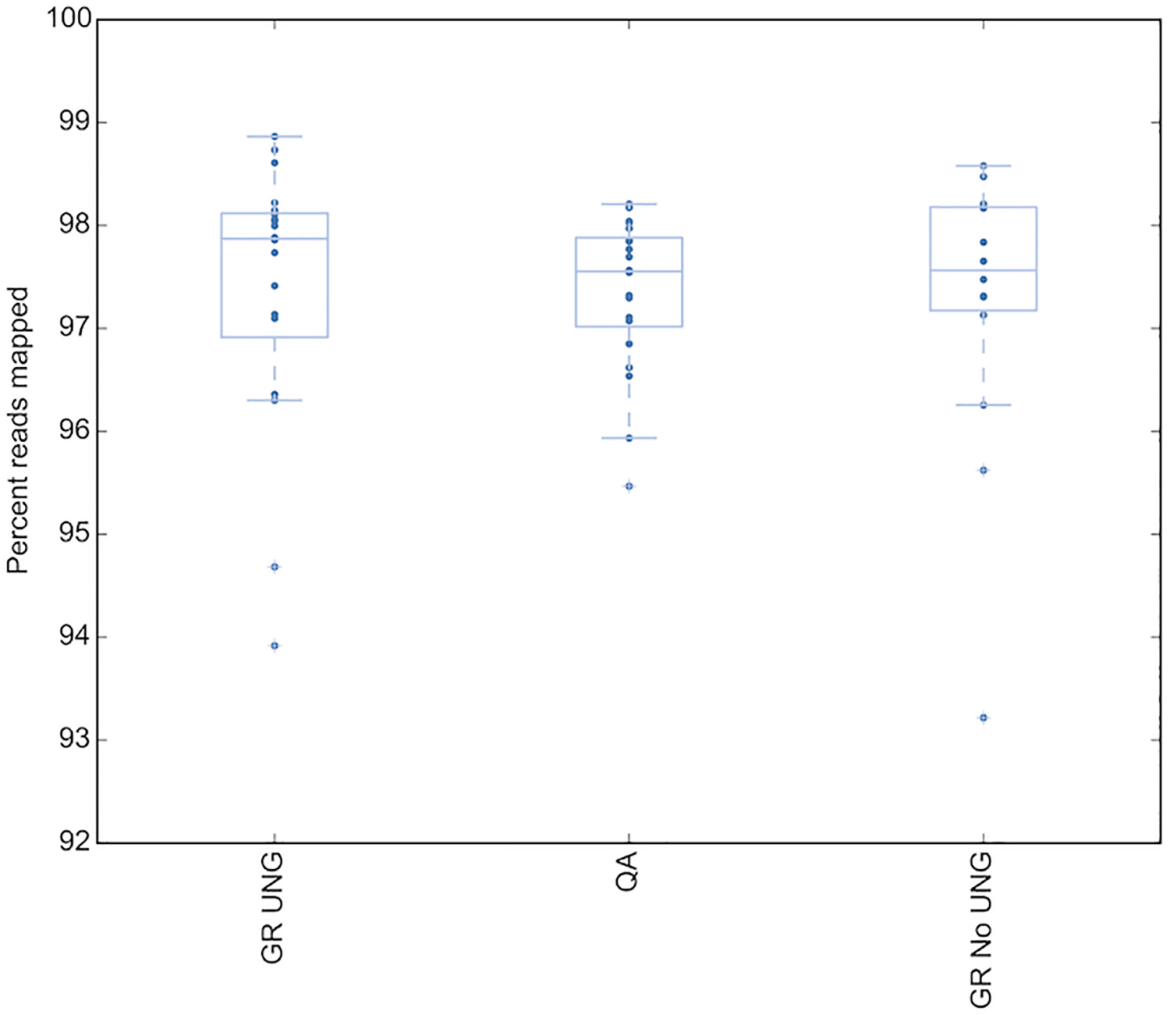
Read quality in the baseline group does not change by DNA extraction type. Read quality as measured by percent reads mapped. GR UNG = GeneRead uracil-N-glycosylase, QA = QiaAmp FFPE kit.

To determine whether any sequencing result differences were due to the effect of the DNA extraction treatment, three different extraction types were assessed across a group of 20 colorectal samples from 2015/2016. The three DNA extraction types were; Qiagen FFPE kit using heat and mineral oil for deparaffization, Qiagen GeneRead Kit with UNG, and Qiagen GeneRead Kit without UNG. Sequencing QC metrics were assessed on all samples for all extraction types, and upon analysis of the sequence no significant difference in raw read count, (P = 0.9) (Fig 2) or read quality as determined by assessing percent reads mapped (P = 0.5) (Fig 3) was observed between DNA extraction method. Thus the DNA extraction process alone is not sufficient to skew the sequencing data quality.

**Fig 4:**
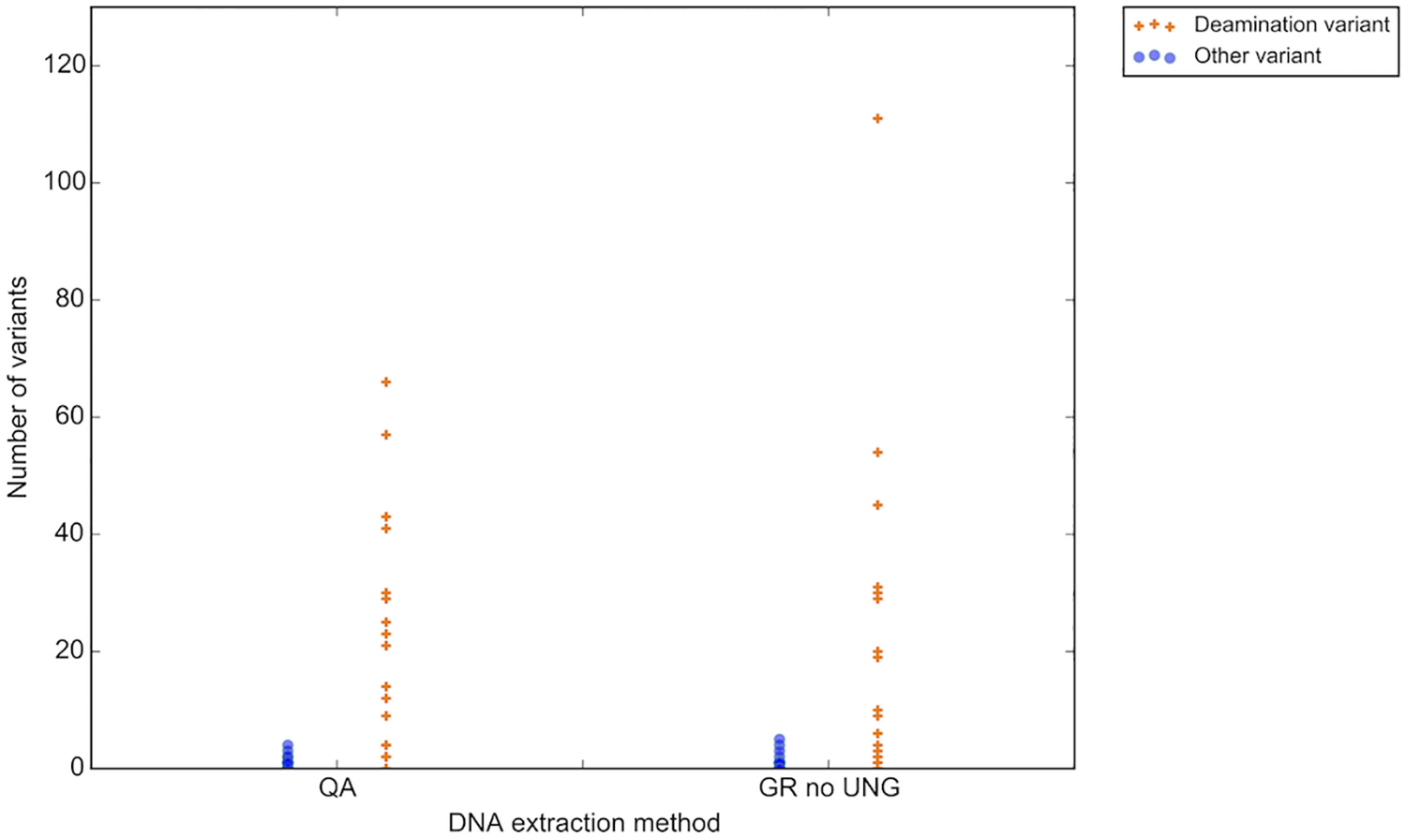
Number of deamination variants ranges widely amongst samples when fixation time is uncontrolled. Orange crosses represent number of deamination variants (y-axis; C->T/G->A). Blue dots represent all other possible variants. GR UNG = GeneRead uracil-N-glycosylase, QA = QiaAmp FFPE kit.

As a baseline of expected deamination events across a group of samples with unknown fixation time, the 20 colorectal samples of similar sample age (no samples older than 2015) were assessed using the three different DNA extraction methods (Fig 4). To account for patient to patient variant count bias, non-UNG treated samples were normalized with the matched UNG treated sample from the same patient. The number of deamination variants per sample ranged from zero to 111, and no discernable difference between the number of deamination variants per sample could be seen between the two extraction kits when UNG was not employed (P = 0.5, (Wilcoxon rank-sum test)).

### Characterization of QC metrics and deamination events across increased formalin fixation time

#### Ground truth somatic mutation profile of the three fixation patients

On assessing the sequencing data from the snap frozen samples, three different somatic mutation profiles became apparent for each of the three patients. The first patient had no somatic mutations, the second patient had a BRAF V600E mutation and the third patient was KRAS G12V positive. The DNA was run in triplicate for all three patients and the VAF was consistent for all runs for the two patients with SNVs; *BRAF* V600E for Patient 2 and *KRAS* G12V for Patient 3 (Table 3).

**Table 3:** Somatic mutation profile.

Patient	Gene	Position	cDNA	Codon	VAF (%)	Routine Genetics
1	WT	WT	WT	WT	WT	MSH2 deleted
2	*BRAF*	Chr7: 140453136	c.1799T>A	V600E	41.09–41.60	BRAF V600E (IHC) MMR intact
3	*KRAS*	Chr12:25398284	c.35G>T	G12V	61.17–61.71	MMR Intact

WT = wild-type

Sequencing studies of *KRAS* are routinely performed in colon cancer patients undergoing systemic therapy as they are a contraindication to *EGFR* targeted therapy [11]. *BRAF* genotypes are often assayed in colon cancer, either by immunohistochemistry or other molecular assays as activating *BRAF* mutations are also a relative contraindication to EGFR targeted therapy [12], and *BRAF* status may play an additional role in Lynch syndrome screening [13].

#### Sequencing QC metrics of the fixation group

Comparing the four different fixation times, there was no discernable difference in the quality or quantity of the library constructed as determined by Qubit or the Agilent bioanalyzer. Nor was there a difference in the total number of raw reads across all formalin fixation treatments as compared to the frozen sample. Assessment of the DNA fragment size via agarose gel revealed the expected DNA smear for the FFPE samples and a sharp high molecular weight band for the frozen samples. There was no discernable difference in fragment smear size amongst the FFPE treated samples (results not shown).

**Fig 5:**
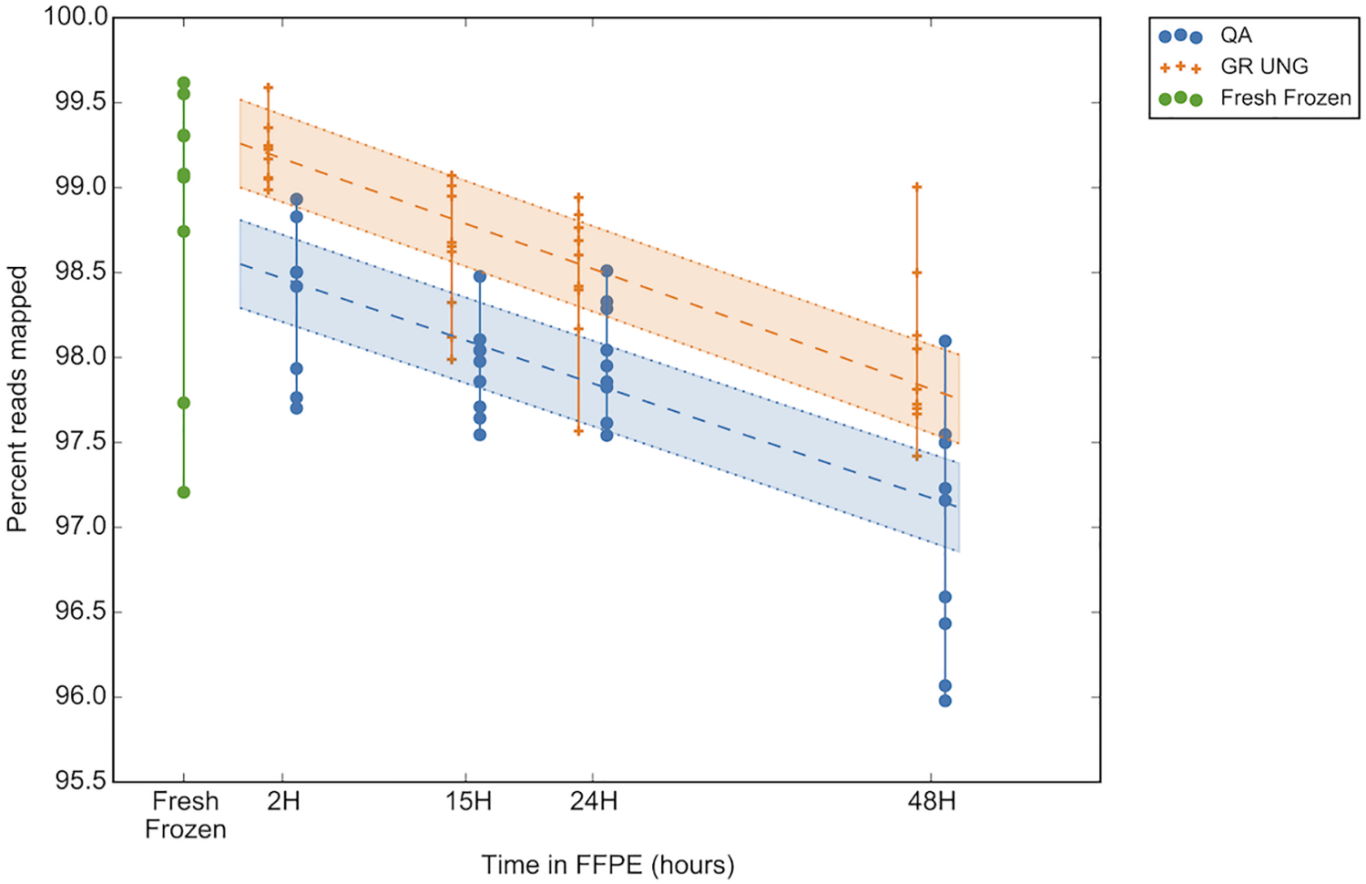
Sequencing read quality decreases over fixation time. Read quality as a measure of percent reads mapped (y-axis) decreases with a longer tissue fixation time (x-axis). Blue dots represent DNA without UNG treatment (extracted using QiaAmp FFPE kit; QA), orange crossed represent matched DNA treated with UNG (extraction using the GeneRead kit; GR UNG). Linear regression line depicted by the central broken line and encompassed by dotted lines representing the 95% confidence interval (CI region colored). Frozen samples used for ground truth are represented by green dots. GR UNG = GeneRead uracil-N-glycosylase, QA = QiaAmp FFPE kit.

Assessment of read quality across fixation times revealed a trend of decreasing read quality with increasing fixation time, as shown by a decrease in percent reads mapped at a rate of approximately -0.3% reads mapped per increasing hour of fixation. This resulted in a change between a median of 98.5% and 99.2% reads mapped for QA and GR UNG respectively at 2 hours fixation versus a median of 97.2% and 97.8% reads mapped for QA and GR UNG respectively at 48 hours fixation (Fig 5). This relationship was significant for both DNA extraction types (P = 0.02 for QA and 0.01 for UNG). Alternatively, this could be indicative of increased noise brought into the reaction over fixation time as the decreased mapped reads equate to more reads being filtered out. Therefore, despite having overall less reads filtered out with UNG, the number of reads filtered out still increases with fixation time so UNG treatment cannot rescue read quality in fixed tissues.

#### Deamination specific mutations assessed over fixation time

**Fig 6:**
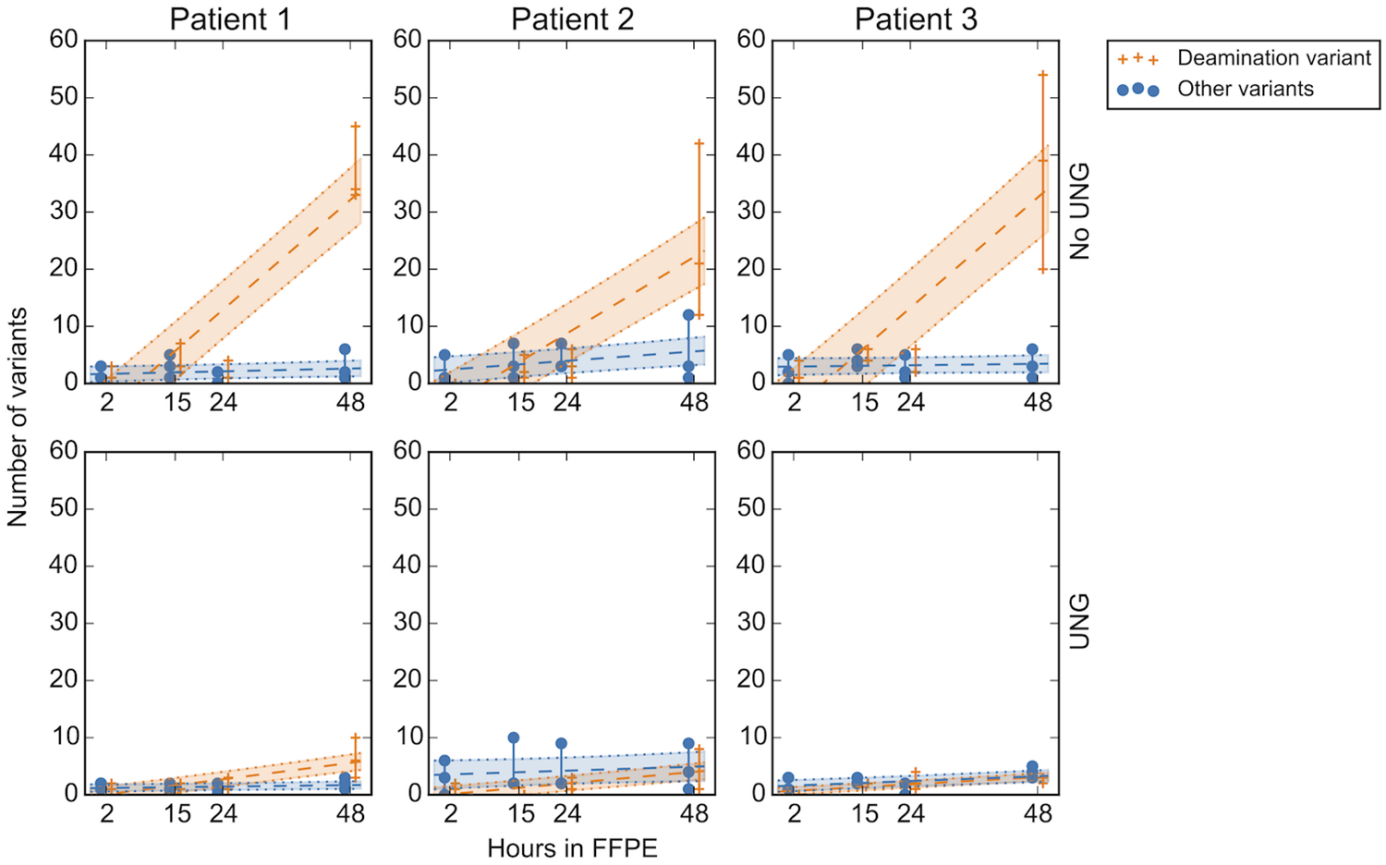
Deamination mutations significantly increase at 48 hours fixation treatment time. Analysis of deamination events in the fixation group for all time points comparing UNG (uracil-N-glycosylase) and non-UNG effects. All variants representative of possible deamination events (C->T, G->A) are denoted by the orange crosses; linear regression line shown by the broken line and encompassed by dotted lines representing the 95% CI (CI regions colored). Similarly all other variants are represented by blue dots, shading and lines. After UNG treatment of the DNA, the deamination events nearly disappear (bottom row).

To determine the possible deamination effects on mutation calling with respect to increased fixation time, DNA extracted from the three patients in the fixation group were treated with UNG. Mutation type was assessed by separating likely deamination mutations (C->T, G->A) from all other possible SNVs. At 48 hours of fixation time, before UNG treatment the deamination SNVs were increased compared to all other mutation types for all three patients across all three replicates (Fig 6). After UNG treatment the number of C/G->T/A events was decreased by a median of 32, 17 and 35 mutations per sample for patients 1, 2 and 3 respectively, supporting the hypothesis that the variants resulted due to deamination from formalin treatment.

### Comparison of sample performance due to archival age

**Fig 7:**
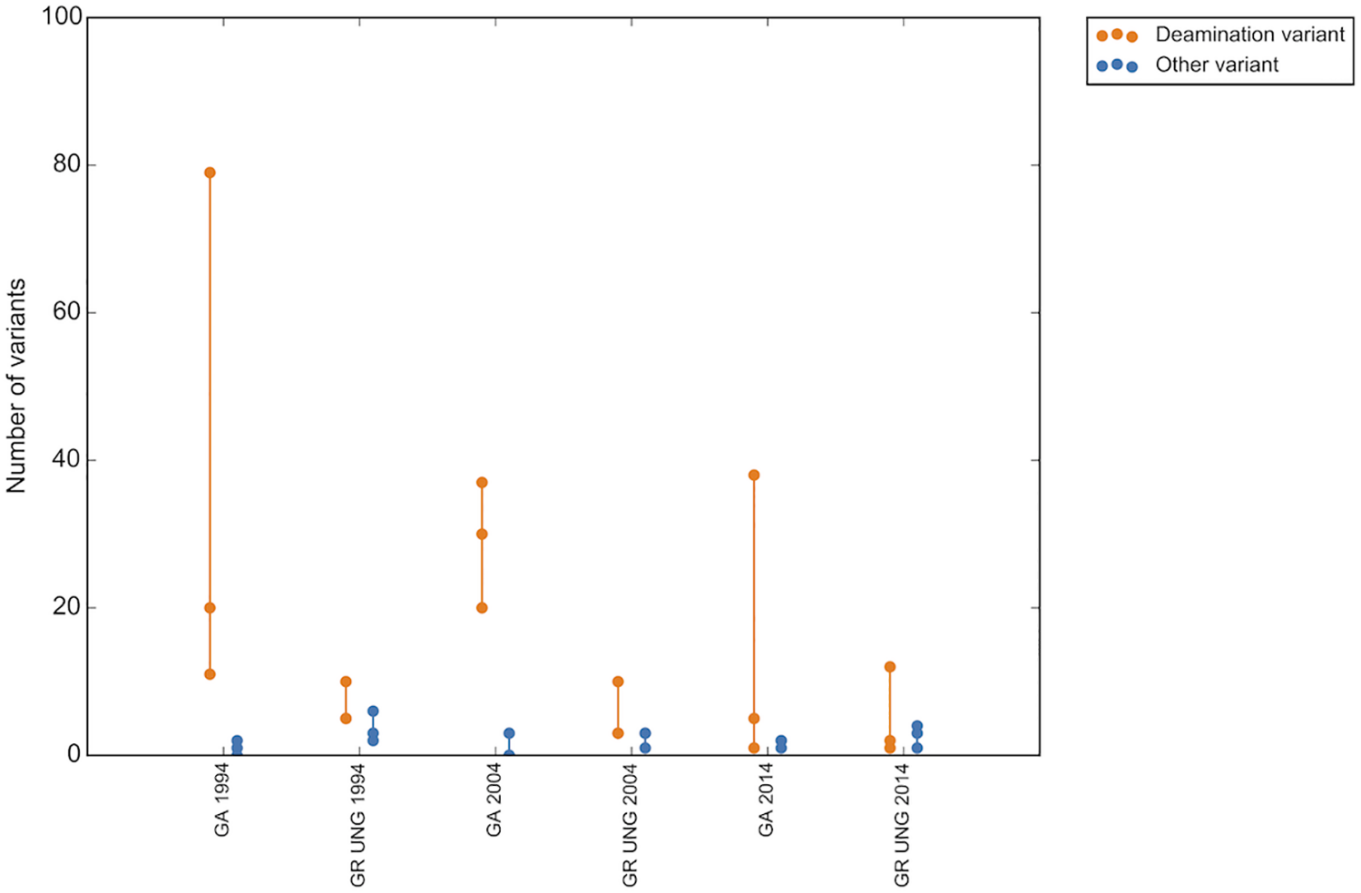
Deamination events increase with sample age when not treated with UNG. GR UNG = GeneRead uracil-N-glycosylase, QA = QiaAmp FFPE kit.

**Fig 8:**
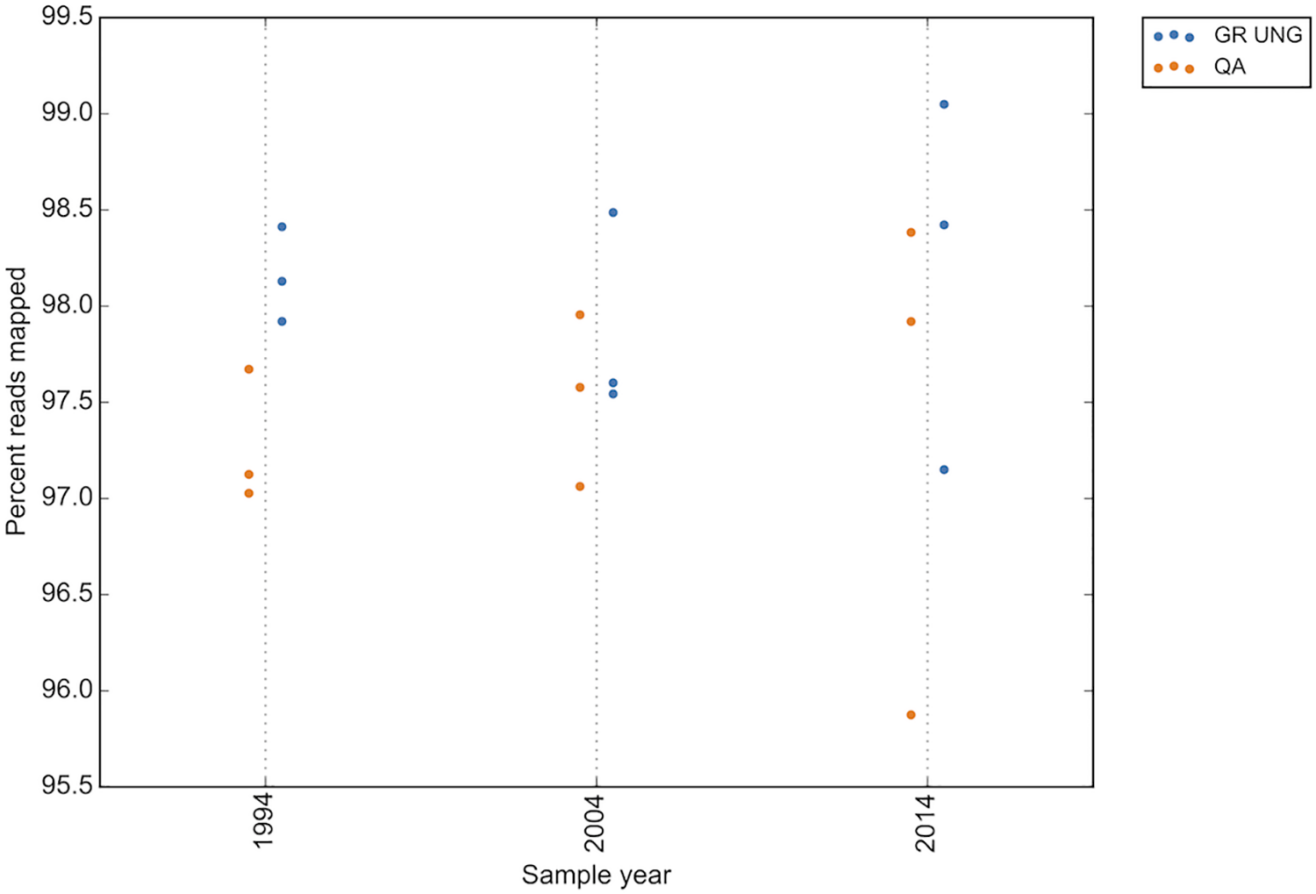
Read quality decreases with sample age in the age group. GR UNG = GeneRead uracil-N-glycosylase, QA = QiaAmp FFPE kit.

As all the samples included in the groups to assess deamination variant status were no older than 2015 (2 years old or less at time of experimental analysis), a separate group of samples was assessed to determine the effect of block age on deamination mutation status (the block age group). A small group of matched tumor/normal samples from 1994, 2004 and 2014 were assessed. Similarly to the 48 hour fixation time, when not treated with UNG, the number of deamination variants increased with sample age as compared to all other mutation types, with a median increase of 15 deamination variants between 1994 and 2014. This effect was partially mitigated with UNG treatment of the DNA prior to sequencing, which reduced the median increase of deamination variants to 3 over the same ten year period (Fig 7). The read quality of older samples also decreases over time, with a median percent reads mapped of 98.4 for UNG treated and 97.9 for untreated samples from 2014 compared to a median of 98.1 for UNG and 97.1 for untreated samples from 1994. This effect is similar to that seen with 48 hour formalin fixation treatment (Fig 8).

### Determination of reported false positive variants

To determine whether a variant is a false positive, analysis is performed using MutationSeq data paired with the patient’s own tissue to omit germline single nucleotide polymorphisms (SNPs). For the baseline and block age groups the matched tissue is the normal FFPE block, but for the fixation group the treatment FFPE sample is paired with the matched frozen tumor that is untreated with formalin. By using tumor tissue as the paired sample for analysis in the fixation group, all true positives are also omitted and only artefacts attributed to the fixation process will be detected.

A true positive was delineated from a false positive if multiple repeated DNA extractions and sequencing consistently detected the same variant. Assessing the fixation group, the highest false positive VAF is 1.48%; thus would not be reported clinically. As samples treated with UNG are claimed to contain fewer false positives, it is interesting to note that the sample containing the highest false positive VAF was UNG treated.

Additionally, due to this paired analysis across multiple sections of tissue in the fixation group, intratumoral heterogeneous (ITH) variants became apparent in two of the three patients. The ITH variants were considered a true positive if they were present in all DNA extractions and repeats for the same FFPE block. If the variant was repeatedly detected in one or a subset of FFPE blocks but not all patient tumor samples, this was indicative of intratumoral heterogeneity (ITH). The false positive and ITH results are listed in S2 Table. Intratumoral heterogeneity is not an uncommon event in colorectal carcinoma and may be a source of variants with low allelic frequency [14]. Thus, when tumor heterogeneity is important to measure, low-prevalence mutations should be verified through sequencing multiple samples from the same paraffin block.

## Discussion

As tumor genotyping is now a staple in clinical oncology practice, tumor sequencing assays must be held to the same standard of laboratory quality as other predictive biomarkers. Formalin fixation has been previously thought to affect patient immunohistochemistry biomarker status, leading to strict guidelines for pre-analytic specimen handling in breast cancer specimens [15, 16].

Here we show that pre-analytic variables including formalin fixation time and block age do have a measurable effect on tumor DNA analysis; however this effect is small and unlikely to affect any clinical outcomes. Next-generation sequencing assays, particularly when performed with deep coverage, are incredibly robust in separating DNA damage from pathologic somatic mutations. The deamination events attributed to formalin fixation in this study occur at a very low frequency and thus would not present a risk of false positive results, as our laboratory uses a minimum threshold of 5% allelic frequency (corresponding to a heterozygous event in a sample with 10% tumor content). Cutoffs in this range are a common practice in molecular pathology and critical in maintaining the validity of sequencing results.

These results suggest that recent FFPE blocks are preferable to older blocks; however clinical indications for testing recent tissue are a more powerful driver than the likelihood of encountering DNA damage to avoid historical tissue samples. The reasons for increased deamination events in older archival tissues are unknown, but may include residual formalin present within the tissues during storage, environmental factors related to the storage facility, or other factors unique to historical tissue processing.

Although these results are reassuring to molecular pathology and clinical oncology practitioners, they do highlight the potential risks associated when working with very low tumor content samples, or reporting very low frequency mutational events. Indeed recently published clinical molecular guidelines in colorectal cancer recommend reporting to 5% VAF and lung cancer to 1% VAF with the ability to detect EGFR T790 in as few as 5% of cells [17, 18]. As our study shows, UNG treatment is not sufficient to eliminate false positive mutations arising from deamination effects in samples for mutations with VAFs <5%, nor does it mitigate poor read quality, with prolonged formalin fixation.

Methylated cytosine, as found at CpG sites, is deaminated directly to thymine, and would not be affected by UNG treatment. Indeed, Do et al. [19] demonstrated that C->T deamination at CpG sites was unchanged after uracil-DNA glycosylase treatment. However our assay is targeted and contains only three potential CpG sites as determined by sequence analysis using UCSC. Looking at variants in these CpG regions only, there were no mutations found in the fixation or baseline groups, and only 2 mutations were found in the age group. These two mutations were found in the non-UNG treated samples, but not in the UNG treatment tissue. Thus for our assay artefacts occurring at methylation sites seems to be a non-issue. However, we would recommend that clinicians employing UNG treatment to patient samples and sequencing larger regions that encompass promoter sites be cognizant of this artefact that cannot be mitigated with UNG treatment.

To most accurately replicate what occurs in the clinic we limited our analysis to FFPE tissues and excluded cell lines, thus we did not include a positive deamination control. Though we see the advantage of using a deamination control, cell lines behave much differently than resected tissue. To ensure true formalin effects were being captured over fixation time, the deamination events were directly compared to their fresh frozen matched tissue.

Patient tissues are not rigorously controlled for a pre-set formalin fixation time. Our results show that samples fixed for 48 hours have increased variants likely arising from the deamination of cytosine during the formalin fixation process. Though these samples can be rescued from deamination artefacts using UNG (which does not rescue read quality), it would be preferable to fix samples for 24 hours or less so that UNG treatment would not be necessary. Similarly, older archived patient samples have increased deamination variants. Although deamination variants did increase with sample fixation time and age, the allelic frequencies remained below 2%, which is below the 5% reporting threshold for the NGS assay used in this study and hence would not have been reported clinically. As tissue fixation habits in the clinic are unlikely to change, it is necessary to establish a more robust method to safely predict mutations at low allelic frequency to ensure patient safety from false positive calls. This could include the use of barcodes to enable the counting of mutated templates in source DNA. With the need for detection of subclonal resistance mutations at low allelic frequencies, this is especially a concern.

## Supporting information

S1 Table Complete mutation list. Mutations used for all analyses are listed for all three patient groups.
(XLSX)

S2 Table False positive mutations. Complete mutation list for all extractions across all fixation times with a reportable score of 0.9 or greater are listed with their corresponding VAF for the fixation group. True somatic mutations are highlighted in orange, ITH mutations are highlighted in green, and false positives are highlighted in pink.
(XLSX)
